# A defined N6-methyladenosine (m^6^A) profile conferred by METTL3 regulates muscle stem cell/myoblast state transitions

**DOI:** 10.1038/s41420-020-00328-5

**Published:** 2020-09-29

**Authors:** Brandon J. Gheller, Jamie E. Blum, Ern Hwei Hannah Fong, Olga V. Malysheva, Benjamin D. Cosgrove, Anna E. Thalacker-Mercer

**Affiliations:** 1 Division of Nutritional Sciences, Cornell University, Ithaca, NY USA; 2 Meinig School of Biomedical Engineering, Cornell University, Ithaca, NY USA; 3 Center for Exercise Medicine, University of Alabama at Birmingham, Birmingham, AL USA; 4 Department of Cell, Developmental and Integrative Biology, University of Alabama at Birmingham, Birmingham, AL USA

**Keywords:** Muscle stem cells, Transcriptomics, Stem-cell differentiation

## Abstract

Muscle-specific adult stem cells (MuSCs) are required for skeletal muscle regeneration. To ensure efficient skeletal muscle regeneration after injury, MuSCs must undergo state transitions as they are activated from quiescence, give rise to a population of proliferating myoblasts, and continue either to terminal differentiation, to repair or replace damaged myofibers, or self-renewal to repopulate the quiescent population. Changes in MuSC/myoblast state are accompanied by dramatic shifts in their transcriptional profile. Previous reports in other adult stem cell systems have identified alterations in the most abundant internal mRNA modification, N6-methyladenosine (m^6^A), conferred by its active writer, METTL3, to regulate cell state transitions through alterations in the transcriptional profile of these cells. Our objective was to determine if m^6^A-modification deposition via METTL3 is a regulator of MuSC/myoblast state transitions in vitro and in vivo. Using liquid chromatography/mass spectrometry we identified that global m^6^A levels increase during the early stages of skeletal muscle regeneration, in vivo, and decline when C2C12 myoblasts transition from proliferation to differentiation, in vitro. Using m^6^A-specific RNA-sequencing (MeRIP-seq), a distinct profile of m^6^A-modification was identified, distinguishing proliferating from differentiating C2C12 myoblasts. RNAi studies show that reducing levels of METTL3, the active m^6^A methyltransferase, reduced global m^6^A levels and forced C2C12 myoblasts to prematurely differentiate. Reducing levels of METTL3 in primary mouse MuSCs prior to transplantation enhanced their engraftment capacity upon primary transplantation, however their capacity for serial transplantation was lost. In conclusion, METTL3 regulates m^6^A levels in MuSCs/myoblasts and controls the transition of MuSCs/myoblasts to different cell states. Furthermore, the first transcriptome wide map of m^6^A-modifications in proliferating and differentiating C2C12 myoblasts is provided and reveals a number of genes that may regulate MuSC/myoblast state transitions which had not been previously identified.

## Introduction

Skeletal muscle regeneration is reliant upon a population of skeletal muscle-specific adult stem cells (MuSCs) and their ability to employ a myogenic program to form new skeletal muscle. In mature skeletal muscle, MuSCs remain in a quiescent state until activated by injury associated ques, at which point they enter the cell cycle, give rise to a transient myoblast population, and divide^1,2^. After a series of cell divisions, a subset of MuSCs will return to the quiescent state to renew the MuSC population that is able to respond to subsequent injuries while another subset of MuSCs will commit to the myogenic lineage and proceed to terminal differentiation^3^. MuSCs that differentiate, will restore damaged skeletal muscle through formation of new myofibers or fusion with existing myofibers. The process of MuSC differentiation and fusion is known to include the choreographed expression of several transcription factors termed the myogenic regulatory factors (MRFs) including myoblast determination protein 1 (*MyoD*), myogenic factor 5 (*Myf5*), and myogenin (*Myog*)^2^. The temporal sequence of the expression of each MRF is well documented however, less is known about how modification of these mRNAs influences their translation and regulatory functions.

Posttranscriptional modifications, particularly the most abundant mRNA modification, N6-methyladenosine (m^6^A), have rapid, pronounced effects on mRNA stability, splicing, and translation, and therefore a cell’s transcriptional profile. m^6^A modification requires a defined set of “writer” proteins including the active methyltransferase, methyltransferase-like 3 (METTL3), to deposit methyl groups on mRNA^4^. Homozygous knockout of *Mettl3* is embryonic lethal in mice^5^. *Mettl3* knockout in embryonic stem cells, however, is viable and has been shown to increase cell proliferation and reduce differentiation capacity when cells are in the naive state^6^. Intriguingly, the opposite effects of *Mettl3* knockout on cell state were observed in primed, pluripotent stem cells^7^. The effect of METTL3 depletion on stem cell state is also dependent on stem cell lineage. For example, reducing m^6^A levels in hematopoietic stem cells reduces proliferation and promotes differentiation^8^ whereas increasing m^6^A levels in adult neural stem cells reduces differentiation^9^. Therefore, m^6^A modifications are central in regulating stem cell state, however, their role is lineage dependent.

To date, only two studies have evaluated the effects of m^6^A modification on myoblasts^10,11^. The first study evaluated the effect of depletion of the m^6^A “eraser,” mRNA demethylase, Fat mass and obesity-associated protein (FTO), in the immortalized myoblast cell line, C2C12 myoblasts. Depletion of FTO led to impaired differentiation and fusion in vitro and impaired postnatal skeletal muscle development in vivo^10^. It is difficult to attribute these effects solely to reductions in m^6^A as FTO has recently been shown to act on m^6^A as well as N6,2-O-dimethyladenosine (m^6^A_m_), a distinct mRNA modification which occurs exclusively near the mRNA cap^12^. The second study used siRNA to knockdown the m^6^A writer, METTL3, which would be expected to have inverse results from knockdown of the m^6^A eraser, FTO, however a reduction in *MyoD* mRNA levels and protein expression of the terminal differentiation marker embryonic myosin heavy chain (eMHC) was observed^11^. Consequently, m^6^A modification appears to be implicated in C2C12 myoblast state transitions, but the directionality of the effect cannot be inferred from the available literature.

Our objective was to determine if m^6^A deposition on mRNA via *Mettl3* is a regulator of MuSC/myoblast state in vitro and in vivo. This study defines the temporal dynamics of m^6^A levels in whole skeletal muscle tissue during the regeneration process, post injury, and in C2C12 myoblasts during in vitro proliferation and differentiation. Using liquid chromatography/mass spectrometry (LC–MS), we identified increased m^6^A levels in skeletal muscle tissue in the early stages of tissue regeneration compared to pre-injury. Furthermore, LC–MS analysis and m^6^A-specific RNA-sequencing (MeRIP-seq) demonstrated increased m^6^A in C2C12 myoblasts during proliferation compared to differentiation. MeRIP-seq of proliferating and differentiating C2C12 myoblasts reveals the first comprehensive profile of mRNAs that are preferentially m^6^A-modified in different myoblast states and reveals that m^6^A modification is closely linked to genes implicated in transcriptional regulation during proliferation. Finally, we show that *Mettl3* knockdown slows proliferation in primary mouse MuSCs and enhances their primary transplant capacity.

## Results

### Global m^6^A levels decline during the MuSC transition from proliferation to differentiation

**Fig. 1:**
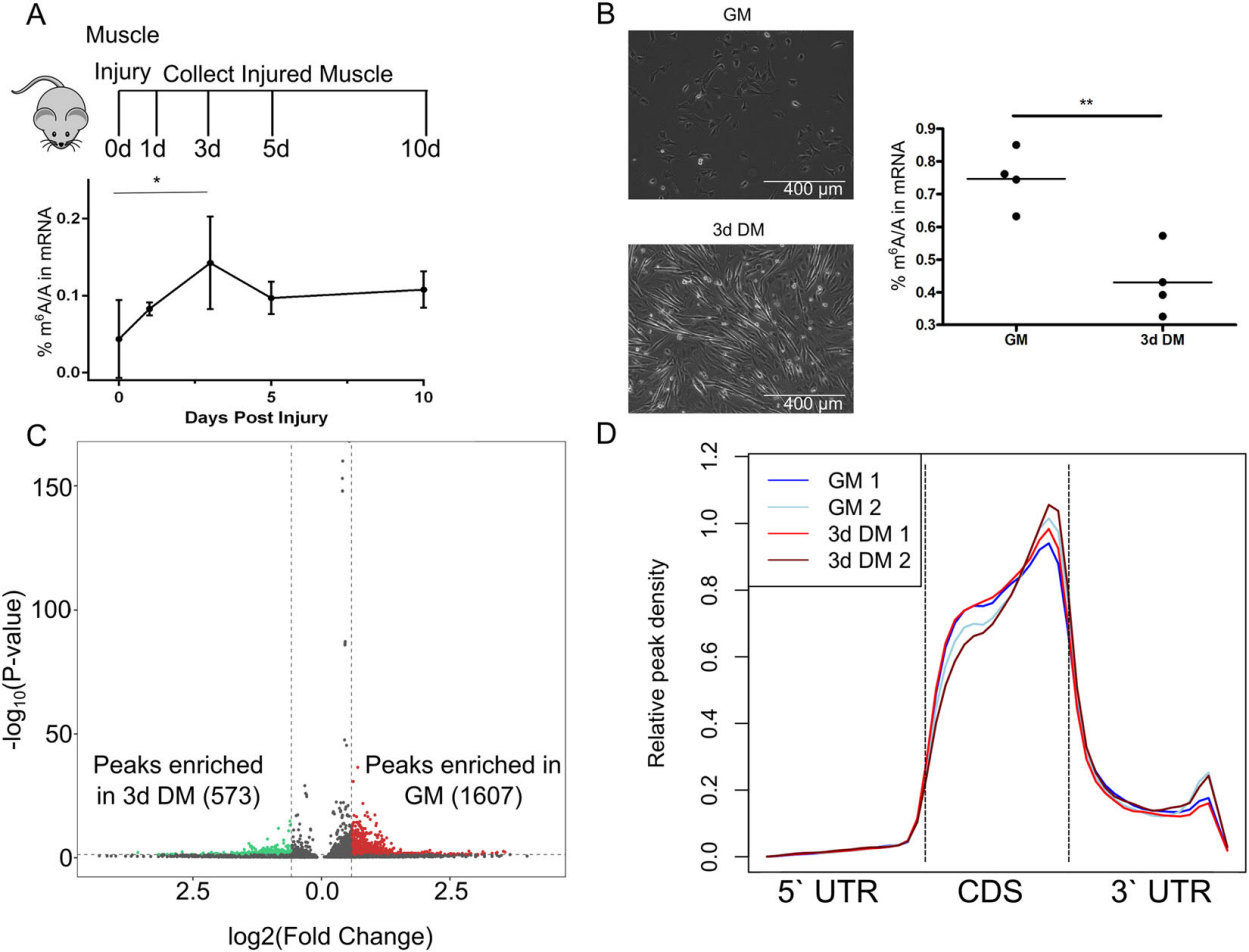
m^6^A-modifications are dynamically regulated in regenerating skeletal muscle and C2C12 myoblasts. **a** The TA of Pax7^nTnG^ mice (*n* = 3–4 per time point) were injected with BaCl_2_, and global m^6^A levels were measured by LC–MS at 0, 1, 3, 5, and 10 dpi. *P < 0.05. **b** mRNA was isolated from C2C12 myoblasts during proliferation (GM) or 3 days after differentiation was induced (3d DM) and global m^6^A levels were measured by LC–MS (*n* = 4). **P < 0.01. **c** Volcano plot displaying unique m^6^A-peaks significantly enriched in C2C12 myoblasts in GM (red, *n* = 2) or 3d DM (green, *n* = 2) according to MeRIP-seq. **d** Distribution of m^6^A peaks along all transcripts identified as m^6^A-modified by MeRIP-seq. Each individual replicate is plotted (GM, *n* = 2; 3d DM, *n* = 2). UTR untranslated region, CDS protein coding region.

Global m^6^A levels were assessed in skeletal muscle tissue after BaCl_2_ induced injury using LC–MS. In whole skeletal muscle tissue, relative m^6^A levels increased at 3 days post injury (dpi) compared to baseline (Fig. 1a). 3 dpi corresponds to a time of substantial MuSC proliferation but also infiltration of a number of other cell types that support the skeletal muscle regenerative process^13–15^. Global m^6^A levels at later times points when MuSC differentiation is initiated (i.e., 5 and 10 dpi) were not different from baseline suggesting this increase in m^6^A levels was specific to MuSC proliferation. To understand the dynamics of global m^6^A levels at the MuSC/myoblast level, during this period of rapid proliferation, C2C12 myoblasts (Fig. 1b) and primary mouse myoblasts (Supplementary Fig. 1A) were used. m^6^A levels were greater when cells were cultured in proliferation favoring media for 24 h (GM) compared to when cells were switched from GM to serum restricted, differentiation inducing media for 3 days (3d DM) in both myoblast models. Because both myoblast models behaved similarly and due to the large amount of input required for downstream applications, C2C12 cells were used for subsequent cell culture experiments, unless otherwise specified.

### MeRIP-sequencing reveals dynamic changes in transcript-specific m^6^A-modification abundance but not the frequency of m^6^A-modification location on transcripts

To map the changes in the transcriptome marked by m^6^A as myoblasts transition from proliferation to differentiation, MeRIP-seq was performed in GM and 3d DM samples. Confirming our LC–MS data, MeRIP-seq revealed an enrichment of m^6^A-modified transcripts in GM (vs. 3d DM) samples (Fig. 1c, Supplementary Tables 1, 2). The distribution of m^6^A across transcripts was similar in GM and 3d DM samples with modifications being most abundant at the boundary of the coding sequence and 3′ regions (Fig. 1d). Therefore, we concluded that a global- and transcript-specific program of m^6^A enrichment is associated with maintaining myoblasts in a proliferative state and that this effect is not driven by differences in the location of m^6^A-modification on transcripts.

### Characterization of the proliferation-specific m^6^A profile at the individual transcript level

**Fig. 2:**
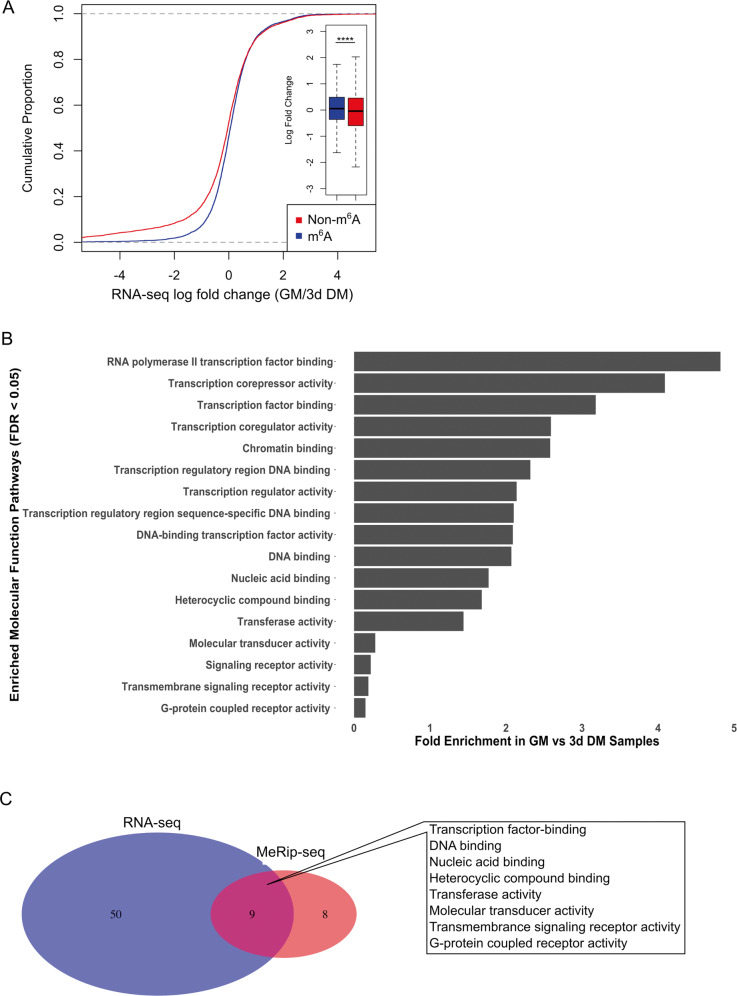
MeRIP-seq analysis identifies transcriptional regulation as the main target of m^6^A-modification during C2C12 myoblast proliferation. **a** The abundance of m^6^A-modified vs. non-m^6^A-modified mRNAs in GM based on RNA-seq comparing GM vs. 3d DM samples. m^6^A-modification was determined by MeRIP-seq. Inset, box plot of the mRNA log fold change (GM/3d DM) of m^6^A-modified and non-m^6^A-modified mRNAs. ****P < 2.2 × 10^−16^ (two-sided Kolmogorov–Smirnov test). **b** Enriched PANTHER GO-slim Molecular Function Pathways identified from m^6^A-enriched genes in GM vs. 3d DM samples. **c** Venn diagram displaying overlap of GO-slim Molecular Function Pathways overrepresented in GM vs. 3d DM samples in both RNA-seq (blue) and MeRIP-seq (red) datasets.

MeRIP-seq analysis revealed 1607 m^6^A peaks representing 862 unique genes enriched in GM compared to 3d DM (Fig. 1c). The top m^6^A-enriched transcripts, based on fold change (GM to 3d DM), were *Dmp1*, *Ccdc73*, *GM5141*, *Zfp316*, *Zfp157*, *Cebpa*, *Gprin1*, *Chst7*, *Ncam1*, and *Nlrx1* (Supplementary Table 1). Comparing the MeRIP-seq analysis with results from concurrently performed bulk RNA-seq (Supplementary Table 3) it was noted that only 14 transcripts were both m^6^A-enriched in GM compared to 3d DM (Supplementary Table 4) and differentially expressed in GM (vs. 3d DM). Of the 14 common transcripts, 7 were zinc finger proteins. To evaluate the effect of m^6^A-modification on individual gene expression, we compared whether having at least one m^6^A-modification predicted relative gene expression (Fig. 2a). Overall, transcripts with at least one enriched m^6^A-modification site in myoblasts during proliferation (i.e., GM samples) were moderately more likely to have increased gene expression; increased expression was determined by fold change >1.0 (GM/3d DM) for transcripts in the RNA-seq dataset (Fig. 2a).

To identify Molecular Function Pathways associated with the m^6^A-modified mRNAs that were overrepresented in GM, we performed PANTHER GO-slim Molecular Function analysis (Fig. 2b, Supplementary Table 5)^16^. An emerging theme from the PANTHER analysis was that m^6^A-modified transcripts that clustered into related Molecular Function Pathways were related to transcriptional regulation (e.g., *transcription factor binding*, *transcription coregulator activity*, and *chromatin binding*). We next performed PANTHER GO-slim analysis on differentially regulated transcripts during GM compared to 3d DM, based on bulk RNA-seq results (Supplementary Table 6); we identified nine overlapping Molecular Function Pathways between the RNA-seq and MeRIP-seq analyses (Fig. 2c and Supplementary Table 7). Similar to our analysis at the single transcript level, the incomplete overlap of affected Molecular Function Pathways between the MeRIP-seq and RNA-seq datasets indicates that some of the functional outcomes of m^6^A-modifications during GM are beyond enhanced transcript stabilization and/or decay.

### Characterization of the differentiation-specific m^6^A profile at the individual transcript level

**Fig. 3:**
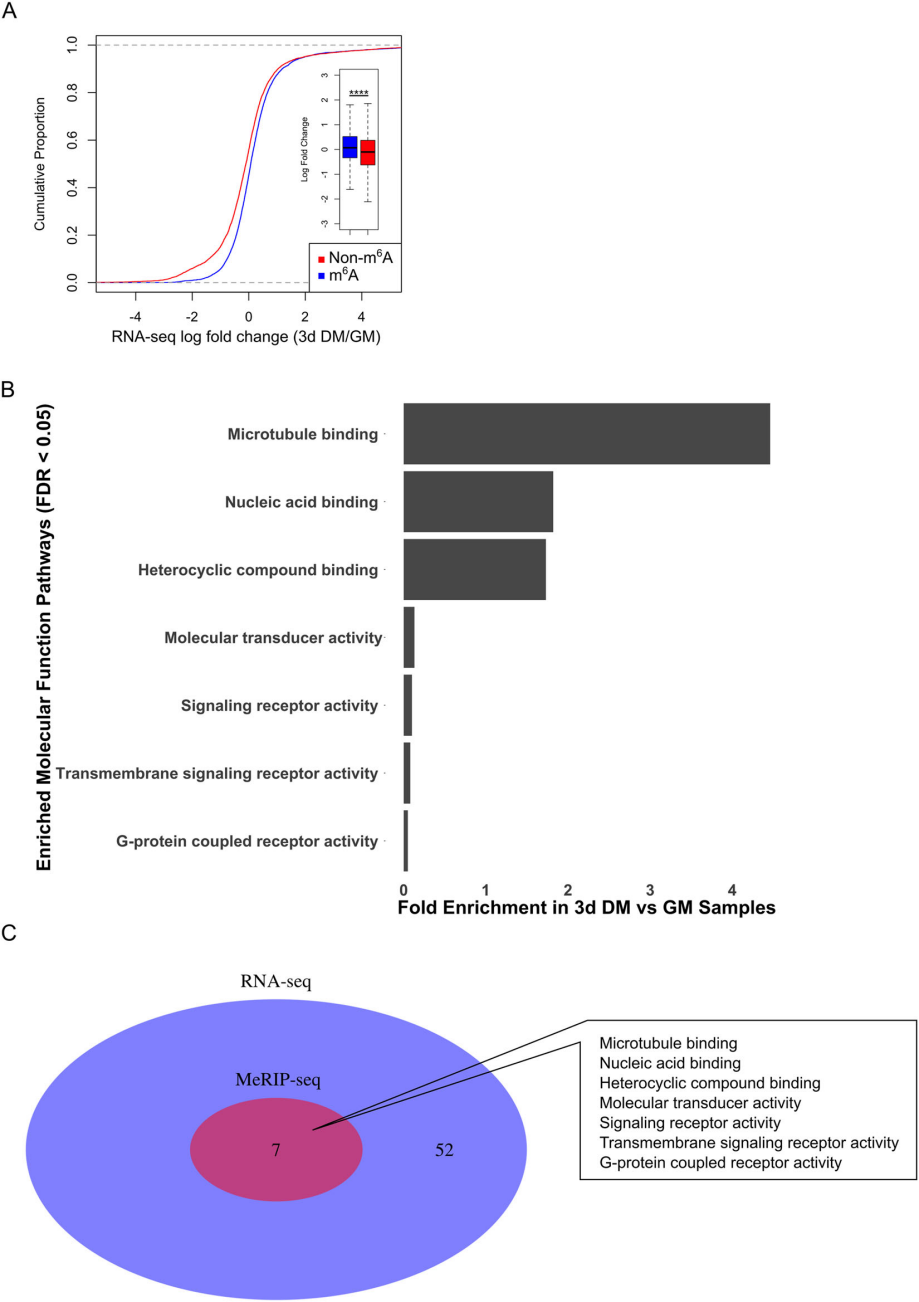
MeRIP-seq analysis identifies microtubule binding as a main target of m^6^A-modification during C2C12 myoblast differentiation. **a** The abundance of m^6^A-modified vs. non-m^6^A-modified mRNAs in 3d DM based on RNA-seq comparing 3d DM vs. GM samples. m^6^A modification was determined by MeRIP-seq. Inset, box plot of the mRNA log fold change (3d DM/GM) of m^6^A-modified and non-m^6^A-modified mRNAs. ****P < 2.2 × 10^−16^ (two-sided Kolmogorov–Smirnov test). **b** Enriched PANTHER GO-slim Molecular Function Pathways identified from m^6^A-enriched genes in 3d DM vs. GM samples. **c** Venn diagram displaying overlap of GO-slim Molecular Function Pathways overrepresented in 3d DM vs. GM samples in both RNA-seq (blue) and MeRIP-seq (red) datasets.

We repeated the analysis for transcripts that emerged as m^6^A-enriched in the 3d DM MeRIP-seq dataset; 573 m^6^A peaks were identified as associated with 340 unique genes that were significantly enriched in 3d DM vs. GM samples (Fig. 1c). Using fold change, the top 10 m^6^A-enriched transcripts in 3d DM were *Flcn*, *Zfp712*, *Zfp59*, *Zfp964*, *Atxn712*, *Gsdme*, *Taf12*, *Col23a1*, *Ankfy1*, and *Cep851* (Supplementary Table 2). Only six transcripts were both differently regulated in the RNA-seq dataset and m^6^A-enriched in 3d DM based on the MeRIP-seq dataset (Supplementary Table 8). Similar to GM, in 3d DM having at least one m^6^A-modification enrichment site present on transcript tended to increase the likelihood that the corresponding transcript was expressed to a greater degree in 3d DM vs. GM (Fig. 3a).

PANTHER GO-slim Molecular Function Pathway analysis revealed fewer Molecular Function Pathways related to transcriptional regulation than appeared in the GM dataset (Figs. 2b, 3b). The most prominent pathway enriched in 3d DM was *microtubule binding* (Fig. 3b and Supplementary Table 8). Furthermore, unlike in GM, all Molecular Function Pathways identified in the MeRIP-seq dataset emerged as enriched in the RNA-seq dataset suggesting a closer link to m^6^A-modification and transcript abundance in 3d DM than in GM (Fig. 3c and Supplementary Tables 5, 9, 10).

### Mettl3 regulates the myoblast transition from proliferation to differentiation

**Fig. 4:**
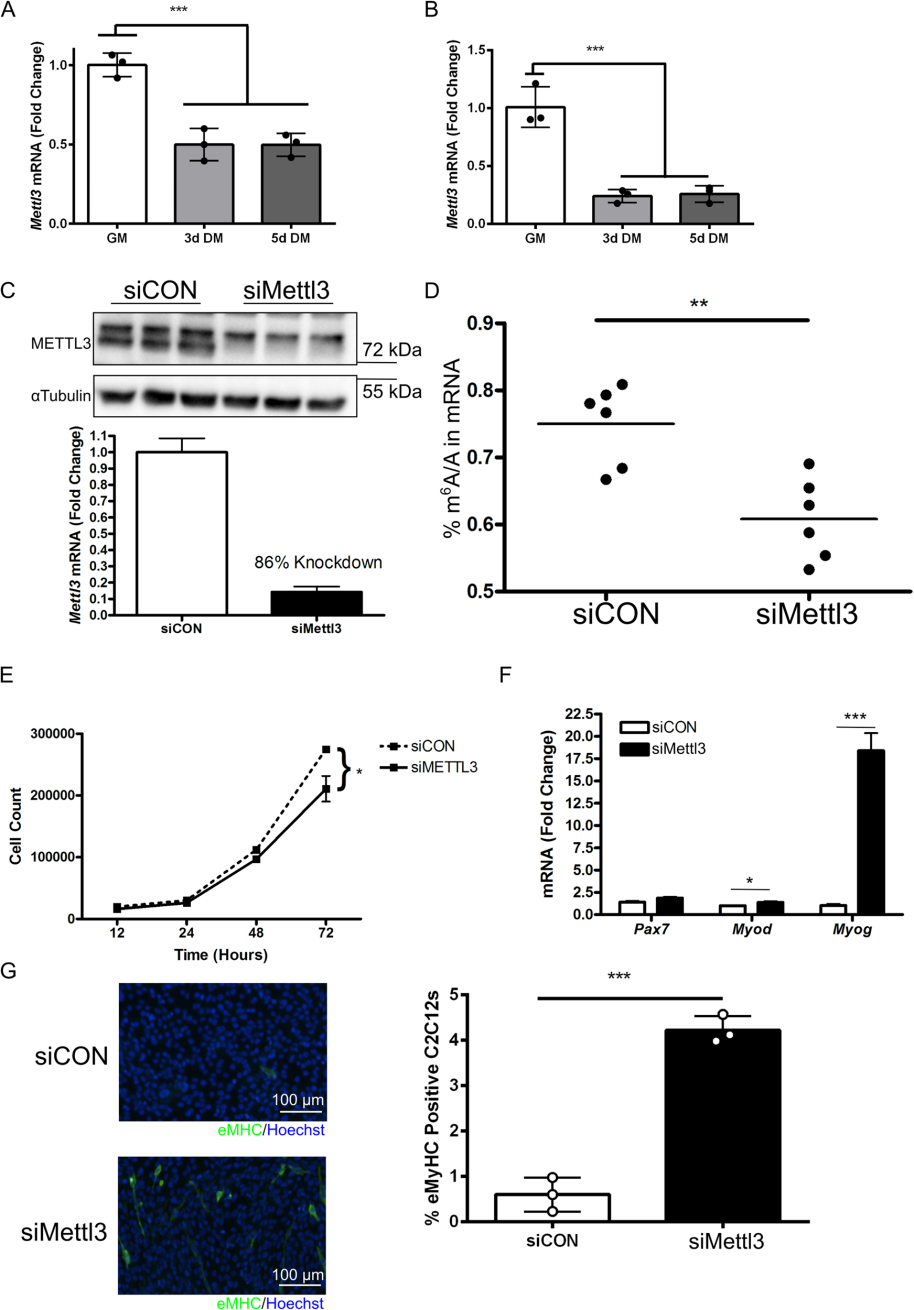
*Mettl3* knockdown forces premature myoblast differentiation. **a** *Mettl3* gene expression levels were assessed via qPCR in C2C12 myoblasts in GM as well 3d and 5d DM (*n* = 3). ***P < 0.001. **b** Gene expression levels of *Mettl3* were measured via qPCR in GM as well 3d and 5d DM in primary mouse myoblasts (*n* = 3). ***P < 0.001. **c** siRNA targeting *Mettl3* was applied to C2C12 myoblasts for 2 days in proliferation favoring media and protein levels (immunoblotting) and gene expression (qPCR) levels of *Mettl3* demonstrated an efficient knockdown (*n* = 3). **d** mRNA was isolated from C2C12 myoblasts after 2 days of siMettl3 or siCON and global m^6^A-modification levels were determined via by LC–MS (*n* = 6). **P < 0.01. **e** Total C2C12 myoblast number was counted at the time points indicated after treatment with siMettl3 or siCON (*n* = 3). *P < 0.05. **f** Gene expression levels of *Pax7*, *Myod1*, and *Myog* were measured via qPCR after 2 days of siMettl3 or siCON (*n* = 3). *P < 0.05; ***P < 0.001. **g** Immunocytochemistry for eMHC in C2C12 cells (left) and quantification (right) after 2 days of siMettl3 or siCON (*n* = 3). ***P < 0.001.

We hypothesized that m^6^A levels were enriched in GM samples due to increased expression of the active m^6^A writer *Mettl3*. To model MuSC activity during regeneration, C2C12 myoblast and primary mouse myoblasts were cultured then prompted to differentiate in vitro by serum restriction; *Mettl3* expression levels markedly declined in both C2C12 myoblast (Fig. 4a) and primary mouse myoblasts (Fig. 4b) after differentiation initiation.

To determine if prevention of m^6^A-modification deposition alone was adequate to initiate differentiation, C2C12 myoblasts were treated with siRNA targeting *Mettl3* (Fig. 4c), which was successful in reducing the frequency of m^6^A-modification (Fig. 4d). Knockdown of *Mettl3* reduced cell count (Fig. 4e). We next evaluated the effect of *Mettl3* knockdown on MRFs associated with proliferation (*Pax7*), early differentiation (*Myod1*), and late differentiation (*Myog*). Gene expression of *Myod1* and *Myog* were significantly increased after *Mettl3* knockdown (Fig. 4f). Finally, *Mettl3* knockdown significantly increased the number of C2C12 myoblasts expressing eMHC, a marker of terminal differentiation (Fig. 4g), indicating a transition from proliferation to differentiation despite cells being cultured in proliferation favoring media. Altogether these data support a model whereby *Mettl3* knockdown in proliferating C2C12 myoblasts reduces global levels of m^6^A-modification and causes premature differentiation of myoblasts.

### Mettl3 knockdown affects primary MuSC engraftment

**Fig. 5:**
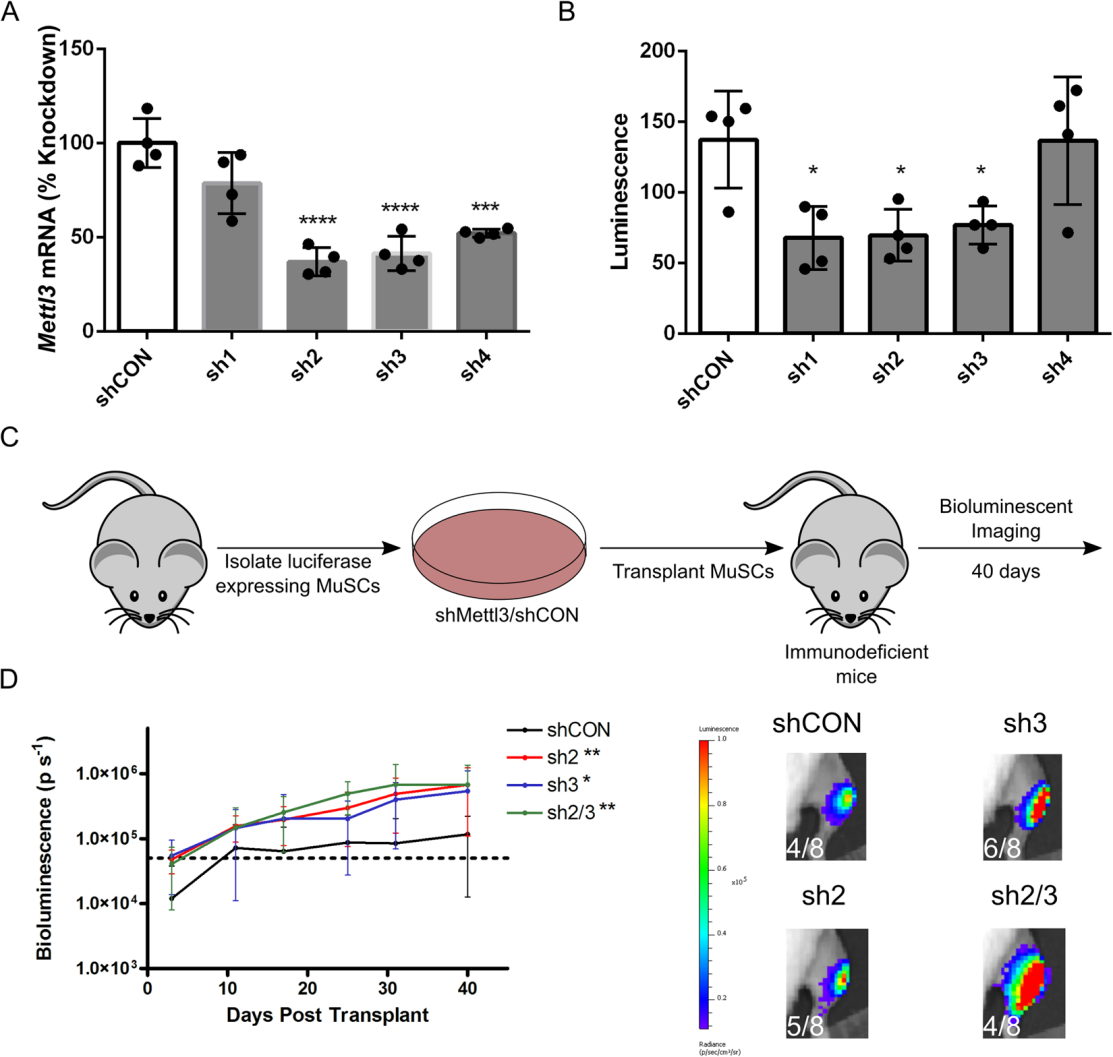
*Mettl3* knockdown enhances MuSC engraftment after primary transplantation. **a** Primary mouse MuSCs were isolated from CMV-*luc* mice, treated with shRNAs targeting *Mettl3* or a nonmammalian control sequence, and cultured for 7 days prior to measuring *Mettl3* gene expression levels via qPCR (*n* = 4). ***P < 0.001; ****P < 0.0001. **b** Luminescence was measured in primary mouse MuSCs after 7 days of culture and treatment with shMettl3 or shCON (*n* = 4). *P < 0.05. **c** Schematic of primary mouse MuSC transplantation strategy; Primary mouse MuSCs were isolated from CMV-*luc* mice and transplanted into the TA of NSG knockout mice (*n* = 8 transplants per condition) and followed for 40 days. **d** Bioluminescence was measured at the time points indicated (left) and representative images are shown with the number of transplants with successful engraftment indicated (right). *P < 0.05; **P < 0.01 (main effect of shRNA, one-way ANOVA).

To test the effect of *Mettl3* knockdown on primary mouse MuSCs, MuSCs were isolated from CMV-*luc* mice, treated with one of four different short-hairpin constructs targeting *Mettl3* (shMettl3) or a nonmammalian control (shCON, Fig. 5a); the two most effective constructs at reducing *Mettl3* levels were used for transplantation experiments. Knockdown of *Mettl3* in primary mouse MuSCs effectively reduced luciferase expression (a surrogate for nuclei number, Fig. 5b) and percent confluence (Supplementary Fig. 2A) after 7 days of culture in a proliferation favoring media. When *Mettl3* was knocked down prior to transplantation the likelihood for engraftment and bioluminescent expression of engrafted MuSCs improved (Fig. 5c, d). Additionally, we performed serial transplants to identify if MuSCs maintained engraftment potential; however, none of the shMettl3 or shCON transplants were capable of engraftment upon secondary transplantation, indicating no MuSCs were retained in a stem like state after primary transplantation (data not shown).

## Discussion

In the experiments described here, we provide the first evidence that global m^6^A levels increase after skeletal muscle injury at a time point corresponding with rapid MuSC proliferation. Additionally, the use of LC–MS m^6^A measurement and MeRIP-seq build upon an earlier report that showed, using the RNA dot blot method, global m^6^A levels in primary mouse myoblasts or C2C12 myoblasts are increased during proliferation and decline during differentiation in vitro^10^. Our MeRIP-seq analysis extended previous findings showing that not only are global m^6^A levels greater in proliferation, but that there are also unique subsets of transcripts which are preferentially methylated in different cell states (i.e., proliferation and differentiation).

The transition from myoblast proliferation to differentiation is accompanied by drastic alterations in the transcriptome^17–19^. Transcriptional changes influencing MuSC/myoblast state transitions have been shown to be affected by a number of processes including changes in DNA and histone methylation of the MRFs^20^. This study provides the first, comprehensive identification of transcripts that are m^6^A-modified in myoblasts during proliferation and in early differentiation. We found no differences in methylation of transcription factors known to drive MuSC state changes such as *Pax7*, *Myod1*, or *Myog* suggesting that the MRFs are not direct targets of dynamic m^6^A-modification but rather, changes in their expression resulting in altered cell state is a secondary effect. Intriguingly, of the top m^6^A-enriched transcripts in GM and in 3d DM, many have not been previously linked to MuSC/myoblast function but have been demonstrated to regulate transcription and even cell state in other cell types. For example, a number of zinc finger proteins including *Zfp316* and *Zfp157* (in GM) as well as *Zfp712*, *Zfp59*, and *Zfp964* (in 3d DM) were among the top ten transcripts differentially m^6^A-enriched during proliferation and differentiation, respectively. While many of the zinc finger proteins that emerged from the MeRIP-seq dataset have not been previously implicated in MuSC/myoblast state transitions, the regulation of myogenic differentiation by other zinc finger proteins is well established^21^. Notably, *Zfp637* was identified as an m^6^A-enriched transcript in GM, compared to 3d DM. *Zfp637* repression is reported to suppress C2C12 myoblast proliferation and its over expression prevents C2C12 myoblast differentiation^22^. Furthermore, the PANTHER GO-slim Molecular Function analysis revealed that 11/17 of the Molecular Function pathways that were overrepresented by m^6^A-enriched transcripts in GM (vs. 3d DM) were related to transcriptional regulation. Therefore, it appears likely that during proliferation, m^6^A-modification is used, in part, to affect the abundance of transcription regulating proteins, such as zinc finger proteins, which in turn define a transcriptional profile favoring cell cycle progression.

A dynamic pattern of *Mettl3* expression during myoblast state changes was observed however, the upstream regulation of this expression remains elusive. m^6^A has been shown to be responsive to a number of external cues such as heat shock^23^ or inflammatory challenges^24^ at the cellular level or restraint stress at the organismal level^25^. During skeletal muscle injury a number of changes to the MuSC microenvironment occur, including cytokine release from invading immune cell populations^26,27^ and release of growth factors from the extracellular matrix^28^. It is likely that one or a combination of these factors is able to repress *Mettl3* expression and drive the reduction in m^6^A levels observed, thereby facilitating the transition from proliferation to differentiation in MuSCs at the correct time.

Transplantation of *Mettl3-*knockdown MuSCs showed improved engraftment capacity compared to controls. Engraftment potential determined by bioluminescence imaging has been identified to be an approximate representation of maintenance of MuSC self-renewal, and therefore stem potential^29^, but is also influenced by the MuSCs’ niche colonization capability^30^. Culturing of MuSCs in vitro prior to transplantation, as was done in this study, is documented to prompt the transition from a stem to a progenitor like state and impair engraftment potential after transplantation^29,31,32^. We hypothesize that this enhanced engraftment capacity of *Mettl3*-knockdown MuSCs is due to increased resistance to activation, and delayed progression toward a progenitor like state, prior to transplantation, resulting in enhanced niche colonization capability. The failure of secondary transplants, from any condition, to engraft supports a loss of stem potential and underscores that the increase in bioluminescent signal observed in Mettl3-knockdown MuSCs was likely due to enhanced niche colonization and not maintenance of a stem like state. A delay to activation of MuSCs due to *Mettl3* knockdown supports a framework where the function of m^6^A-modification in MuSCs is to regulate transitions between cell states including quiescence to activation as well as proliferation to differentiation.

In conclusion, we elucidate METTL3-mediated m^6^A regulation as an important regulator of MuSC/myoblast state transitions and provide the first comprehensive map of m^6^A-modified transcripts during myoblast proliferation and differentiation. Future investigation into the biological function of the genes from the datasets developed here may glean new insight into the earliest stages of terminal differentiation commitment in MuSC/myoblasts. Furthermore, an understanding of the upstream signal that leads to the decline in *Mettl3* transcription upon differentiation initiation would be invaluable in understanding how MuSC-microenvironment communication underlies transcriptional changes that regulate the initial steps in myogenic differentiation.

## Materials and methods

### Mice and animal care

All mice were maintained according to the Guide for the Care and Use of Laboratory Animals and all procedures were approved by The Cornell University Institutional Animal Care and Use Committee. For skeletal muscle injury experiments, previously published B6.Cg-Pax7^tm1(Cre/ERT2)Gaka^/J mice^33^ (JAX: 017763) were crossed with B6N.129S6-Gt(ROSA)26Sor^tm1(CAG-tdTomato*,−EGFP)/Ees^/J mice^34^ (JAX: 005304) to produce the following mouse: Pax7^CreERT2^;Rosa26^nTnG^ (hereafter referred to as Pax7^nTnG^). Pax7^nTnG^ mice were used for all muscle injury experiments. Mice ubiquitously expressing luciferase, FVB-TG(CAG-luc,-GFP)L2G85Chco/J (JAX: 008450, CMV-*luc* mice)^35,36^, were used for the isolation of donor cells for transplantation experiments and were transplanted into immunodeficient NSG mice (NOD.Cg-Prkdc^scid^Il2rg^tm1Wjl^/SzJ, JAX: 005557)^37,38^. Animals were randomly assigned to treatment groups and the treatment group of samples derived from each animal were blinded to the investigator performing downstream analyses. The number of animals per experimental condition are reported in the figure legends and sample sizes were based on convention in the field.

### Skeletal muscle injury

To induce skeletal muscle injury, 50 µL of 1.2% BaCl_2_ (Sigma, St. Louis, MO, USA) was injected into the tibialis anterior (TA) muscle of Pax7^nTnG^ mice under isoflurane anesthesia. Mice were sacrificed at 0 (baseline), 1, 3, 5, and 10 dpi and the TA muscle was snap frozen in liquid nitrogen and stored at −80 °C until mRNA isolation.

### C2C12 myoblast culture

C2C12 myoblasts (ATCC, Manassas, VA, USA) were cultured in a growth medium containing high glucose Dulbecco’s Modified Eagle Medium (DMEM, Gibco, Gaithers MD, USA) supplemented with 10% fetal bovine serum, 4 mM Glutamax (Gibco, Gaithers MD, USA), and 1% penicillin–streptomycin (Corning, Pittston, PA, USA). Cultures were not tested for mycoplasma contamination. When cultures were between 80-100% confluent they were switched to a media containing high glucose DMEM supplemented with 2% heat-inactivated horse serum to promote differentiation. GM samples were taken after 24 h of culture in growth medium when cultures were ~50% confluent and DM samples were taken at various days after the switch to differentiation media, as indicated. All C2C12 myoblasts were used between passage 5 and 10 and were cultured in a 5% CO_2_ atmosphere at 37 °C on collagen (Type I, Rat Tail, Corning, Pittston, PA, USA) coated plates. As per convention in the field, all experiments were repeated at least three times for all cell culture experiments (excluding MeRIP-seq experiments) with specific values noted in the figure legends.

### Primary mouse myoblast culture

Passage 17–20 primary mouse myoblasts, obtained as previously described, were used^39^. Primary mouse myoblasts were cultured in a growth medium of 43% low glucose DMEM (Gibco, Gaithers MD, USA), 40% Ham’s F10 (Gibco, Gaithers MD, USA), 15% fetal bovine serum, 1% penicillin–streptomycin, 1% Glutamax, and 2.5 ng/mL basic fibroblast growth factor (Promega, Madison, WI, USA). When cultures were between 80 and 100% confluent, they were switched to a media containing 2% heat-inactivated horse serum. Similar to C2C12 myoblast experiments, GM samples were obtained at ~50% confluency (24 h after seeding) and DM samples at various days after the switch to differentiation media. Primary mouse myoblasts were seeded on collagen (Type I, Rat Tail) coated plates and maintained in a 5% CO_2_ atmosphere at 37 °C.

### m^6^A LC–MS

For analysis of m^6^A levels in skeletal muscle tissue, RNA was isolated using Trizol reagent (Ambion, Austin, TX, USA) according to the manufacturer’s instructions. For analysis of m^6^A levels in cell lysates and RNA isolated from skeletal muscle tissue mRNA was isolated via Dynabeads Oligo(dT)_25_ (Thermo Fisher Scientific, Waltham, MA, USA) as recommended by the manufacturer. The sample preparation and LC–MS method protocol was adapted from a previously published protocol^40^. One hundred nanograms of mRNA was incubated with 2 U nuclease P1 (Sigma, St. Louis, MO, USA) in a buffer of DEPC treated 25 mM NaCl and 2.5 mM ZnCl_2_ at 37 °C for 60 min to isolate individual nucleotides followed by incubation for 60 min at 37 °C with 0.5 U alkaline phosphatase (Roche, Basel, Switzerland) to remove all phosphate groups, thus producing nucleosides. Samples were then diluted to 200 µL with Di H_2_O. Adenosine (Sigma, St. Louis, MO, USA) and m^6^A (Toronto Research Chemicals, North York, Canada) standard curves were also prepared by serial dilution in Di H_2_O. Prior to injection into the LC/MS/MS system equipped with Accela autosampler and pump (Thermo Fisher Scientific, Waltham, MA, USA) and TSQ Quantum Access (Thermo Fisher Scientific, Waltham, MA, USA), samples and calibration points were mixed with an equal volume solution containing 10 µM of ^13^C_5_-Uridine (Toronto Research Chemicals, North York, Canada) in 0.1% formic acid in Di H_2_O as an internal standard. Ten microliters of sample was injected into a Phenomenex Luna C18 column (250 mm × 4.6 mm, 5 µm, Phenomenex, Torrance, CA, USA). An elution gradient of 0.1% formic acid in Di H_2_O and 0.1% formic acid in methanol at 0.45 mL/min was run as follows: 0 min, 10% methanol; 5 min, 99% methanol; 7 min, 99% methanol; 8 min, 10% methanol; 12 min, 10% methanol. XCalibur (Thermo Fisher Scientific, Waltham, MA, USA) software was used for quantification of m^6^A and adenosine. Multiple reaction monitoring (MRM) transitions in positive mode were: *m*/*z* 282–150 for m^6^A and *m*/*z* 268–136 for adenosine. The m^6^A to total adenosine (m^6^A + total adenosine) peak area ratio was calculated and differences in ratios reported.

### MeRIP-sequencing

RNA for MeRIP-seq was isolated from C2C12 myoblasts using the Omega E.Z.N.A.^®^ Total RNA Kit I (Omega Bio-Tek Inc, Norcross, GA, USA). RNA quality was determined by denaturing agarose gel electrophoresis. mRNA was isolated from >120 µg total RNA using Arraystar Seq-Star^TM^ poly(A) mRNA Isolation Kit (ArrayStar Inc, Rockville, MD, USA) as per the manufacturer’s directions. Isolated mRNA was fragmented to a median size of 10 nt in 20 µL of a buffer containing 10 mM Zn^2+^ and 10 mM Tris-HCl (pH 7.0) for 7 min at 94 °C. Fragment size was confirmed by agarose gel electrophoresis. Ten percent of fragmented mRNA was saved for RNA-sequencing to serve as input control for MeRIP-seq. The remaining fragmented mRNA was used for m^6^A immunoprecipitation.

m^6^A immunoprecipitation was performed by combining fragmented mRNA with 2 µg anti-m^6^A antibody (Cat: 202 003, Synaptic Systems, Goettingen, Germany) and rotated head over tail for 120 min at 4 °C in 500 µL total volume. Twenty microliters of Dynabeads^TM^ M-280 sheep anti-rabbit IgG (clone 11204D, Thermo Fisher Scientific Waltham, MA, USA) were blocked with 0.5 mg/mL bovine serum albumin at 4 °C for 120 min. The blocked Dynabeads and fragmented mRNA/antibody mixture were combined for 120 min at 4 °C. The solution was then washed 3× with a buffer of 10 mM Tris-HCl (pH 7.4), 150 mM NaCl, and 0.1% NP-40 followed by 2× washes with a buffer comprised of 10 mM Tris-HCl (pH 7.4), 50 mM NaCl, and 0.1% NP-40. mRNA immunoprecipitated to the Dynabeads^TM^ was separated by incubation with 200 µL of an elution buffer [10 mM Tris HCl (pH 7.4) 1 mM EDTA, 0.05% SDS, 40 U proteinase K (Qiagen, Hilden, Germany)] for 30 min at 50 °C. mRNA was then extracted by phenol–chloroform and ethanol precipitated before sequencing.

The KAPA Stranded mRNA-seq Kit (Illumina, San Diego, CA, USA) was used to prepare the RNA-seq libraries for m^6^A immunoprecipitated mRNA and input control mRNA libraries. Quality of the libraries was determined using an Agilent 2100 Bioanalyzer. The HiSeq 3000/4000 PE Cluster Kit (Illumina, San Diego, CA, USA) was used to generate clusters from the prepared libraries following the manufacturer’s instructions. The clustered libraries were sequenced on an Illumina HiSeq 4000 system.

### MeRIP-sequencing analysis

The 5′, 3′-adaptor trimmed reads were aligned to the mm10 reference genome using HISAT2 (v2.1.0)^41^. ExomePeak was used for MeRIP peak calling and statistically significant MeRIP enriched regions (MeRIP peaks) were identified at the sample level with a significance threshold of *P* ≤ 0.05^42^. ExomePeak was also used to compare transcripts which were differentially m^6^A-modified between the GM and 3d DM samples with significance set at *P* ≤ 0.05^42^.

### RNA-sequencing analysis

The input control RNA-sequencing results were subjected to differential gene expression analysis using *edgeR*^43^ in RStudio (Version 3.6.1). Genes with at least two counts per million in at least three of the samples were kept for analysis.

### Pathway analysis

Pathway analysis was conducted using the PANTHER (version 14.1) GO-Slim Molecular Function Overrepresentation Test with a false discovery rate (FDR) correction^16^. PANTHER analysis was performed using lists of m^6^A-modified transcripts that were enriched (*P* < 0.05) in GM and separately on lists of transcripts that were enriched in 3d DM based on the MeRIP-seq dataset. PANTHER analysis was also performed using the list of transcripts that were differentially regulated between GM and 3d DM according to the RNA-seq data (FDR < 0.05, log fold change >1.5 or <−1.5).

### C2C12 myoblast siRNA transfection

C2C12 myoblasts were reverse transfected with siRNA-lipid complexes (RNAiMAX, Invitrogen, Carlsbad, CA, USA) targeting *Mettl3* (siMettl3, Cat: 4457298, Ambion, Austin, TX, USA) or a non-targeting control (siCON, Cat: 4390846, Ambion, Austin, TX, USA). C2C12 myoblasts were maintained in growth medium for 48 h after transfection prior to analysis of gene expression, protein expression, and global m^6^A levels.

### Quantitative RT-PCR

The Applied Biosystems High-Capacity cDNA Reverse Transcription Kit (Applied Biosystems, Foster City, CA, USA) was used to synthesize cDNA via reverse transcription of 2 µg RNA extracted using Omega E.Z.N.A.^®^ Total RNA Kit I (Omega Bio-Tek Inc, Norcross, GA, USA) as per the manufacturer’s directions. RNA quality and quantity were determined spectrophotometrically. Quantitative RT-PCR (qPCR) was used to measure mRNA levels of *Mettl3* (Mm01316319), *Pax7* (Mm0135484), *Myod1* (Mm00440387), and *Myog* (Mm00446195). Expression levels were normalized to *18S* (Mm03928990) expression using the Taqman Gene Expression System (Applied Biosystems, Foster City, CA, USA).

### Immunoblotting

Cell lysates were collected using RIPA buffer containing protease (cOmplete, Roche, Basel, Switzerland) and phosphatase (PhosSTOP, Roche, Basel, Switzerland) inhibitors. Following cell lysis, the protein fraction was cleared of other cellular debris via centrifugation (12,000 × *g*, 15 min, 4 °C). Ten micrograms of protein, as determined by the bicinchoninic acid assay (Thermo Fisher Scientific Waltham, MA, USA), was loaded on 10% SDS gels and transferred to PVDF membranes. Membranes were blocked with a chemiluminescent blocking buffer (bl∅k^TM^—CH, MilliporeSigma, Burlington, MA, USA) for 60 min at room temperature (RT) before being transferred into a solution containing either a METTL3 primary antibody (1:1000 dilution in chemiluminescent blocking buffer, overnight incubation at 4 °C, Cat: 15073-1-AP, Proteintech Group Inc, Rosemont, IL, USA) or an HRP-conjugated ɑ-TUBULIN primary antibody (1:1000 dilution in chemiluminescent blocking buffer, 60 min incubation at RT, Cat: 9099, Cell Signaling, Danvers, MA, USA). Membranes incubated with the METTL3 antibody were then washed 3 × 5 min in 0.1% Tween in tris-buffered saline following a 60 min incubation in goat anti-rabbit secondary antibody (1:100,000 dilution in chemiluminescent blocking buffer, Cat: SA00001-2, Proteintech, Rosemont, IL, USA,). Membranes were then incubated in SuperSignal^TM^ West Femto (Thermo Fisher Scientific, Waltham, MA, USA) for 1 min and visualized on the Bio-Rad ChemiDoc MP. METTL3 protein was normalized to α-TUBULIN expression using the ImageLab 4.1 software (Bio-Rad, Hercules, CA, USA).

### C2C12 myoblast population expansion assay

To determine the effect of *Mettl3* knockdown of cell number, C2C12 myoblasts that were cultured in 6-well cell culture dishes were counted using the Moxi Z Mini Automated Cell Counter (ORFLO Technologies, Ketchum, ID, USA) 12, 24, 48, and 72 h after seeding and siRNA transfection in growth medium.

### Immunocytochemistry

Immunocytochemistry was used to determine expression of eMHC. C2C12 myoblasts were fixed for 20 min in 4% paraformaldehyde at RT before 3 × 5 min washes in PBS. Fixed, C2C12 myoblasts were blocked for 60 min at RT in 5% goat serum then incubated with primary eMHC antibody supernatant (DSHB Hybridoma Product F1.652, F1.652 was deposited to the DSHB by Blau, Helen M.)^44^ overnight at 4 °C. C2C12 myoblasts were then washed 3 × 5 min in PBS and incubated in secondary antibody (Alex Fluor^®^ 488 goat anti-mouse, Cat: A11029, Thermo Fisher Scientific, Waltham, MA, USA) diluted 1:1000 in 1% goat serum. Hoechst 33342 was used as a counterstain to identify nuclei and stained cells were imaged on the Celigo S Imaging Cytometer (Nexcelom Bioscience, Lawrence, MA, USA).

### Primary mouse MuSC isolation

Primary mouse MuSC isolation was performed as previously described^31^. CMV-*luc* mice were anesthetized with isoflurane followed by cervical dislocation prior to harvesting of all hindlimb muscles. Muscles were incubated in a digestion cocktail of low glucose DMEM and 2.5 mg/mL Collagenase D (Roche, Basel, Switzerland) for 15 min at 37 °C. Physical dissociation was then performed using the GentleMACS system (Miltenyi Biotec, Bergisch Gladbach, Germany), followed by the addition of Dispase II (0.04 U/mL final concentration stock, Roche, Basel, Switzerland), and an additional 40 min incubation at 37 °C. Further physical dissociation of the muscle tissue was performed using an 18-gauge needle. Cell suspensions were passed through a prewashed 100 µM filter, then a 40 µM filter to remove debris. The remaining cells were pelleted via centrifugation (700 × *g*, 7 min, 4 °C) and resuspended in red blood cell lysis buffer (IBI Scientific, Dubuque, Iowa, USA) for 5 min at RT. The cell suspension was pelleted again, resuspended in 1 mL of a cocktail of biotin conjugated primary antibodies specific for CD45 (2 µL, clone 30-F11, Biolegend, San Diego, CA, USA), CD31 (5 µL, clone 390, Biolegend, San Diego, CA, USA), CD11b (5 µL, clone M1/70, Biolegend, San Diego, CA, USA), and Sca1 (5 µL, clone D7, Biolegend, San Diego, CA, USA), and incubated, on ice, for 25 min. Streptavidin microbeads (12 µL, Miltenyi Biotec, Bergisch Gladbach, Germany) were added to the cell suspension/antibody cocktail mixture and incubated, on ice, for 10 min. After incubation the mixture was passed through prewashed magnetic columns (Miltenyi Biotec, Bergisch Gladbach, Germany). Cells were pelleted and resuspended in a 1 mL solution containing antibodies specific for the following proteins: CD34 (eFluor450, clone ram34, Invitrogen eBioscience, San Diego, CA, USA) α7-integrin (AlexaFluor647, clone r2f, Ablab, British Columbia, Canada), and streptavidin (PE-Cy7, Biolegend, San Diego, CA, USA). After a 20 min incubation on ice, the cell suspension was washed, pelleted, and resuspended in a 200 µL 1:100 solution of propidium iodide. CD34^+^/α7-integrin^+^/CD45^−^/CD31^−^/CD11b^−^/Sca1^−^ cells were isolated via flow cytometry. Isolated primary mouse MuSCs were then seeded on plates coated with laminin (20 µg/mL, Sigma, St. Louis, MO, USA) and RetroNectin^®^ Recombinant Human Fibronectin Fragment (15 µg/mL, Takara Bio USA, Mountainview, CA, USA) in the same growth medium used for primary mouse myoblast culture.

### Primary mouse MuSC shRNA transfection

For in vitro experiments primary MuSCs from CMV-*luc* mice were transfected with short-hairpin lentiviruses targeting *Mettl3* (sh1, clone TRCN0000039110; sh2, clone TRCN0000039111; sh3, clone TRCN0000039112; sh4, clone TRCN0000039113, MISSION, Sigma, St. Louis, MO, USA) or a nonmammalian targeting control (shCON, Cat: SHC002, MISSION, Sigma, St. Louis, MO, USA) 12 h after seeding. Fresh growth medium was replaced 48 h after transfection and continued to be changed every 48 h for 7 days.

For transplantation experiments primary MuSCs from CMV-*luc* mice were transfected with shCON, sh2, sh3, or a combination of sh2 and sh3 (sh2/3) 12 h after seeding. Twenty-four hours following transfection MuSCs were harvested for transplantation.

### Primary mouse MuSC transplantation

For primary transplantation experiments primary MuSCs isolated from CMV-*luc* mice from each shRNA condition (shCON, sh2, sh3, and sh2/3) were individually resuspended in PBS at a concentration of 175 MuSCs per 20 µL PBS 24 h after transfection in culture. NSG knockout mice were anesthetized with isoflurane and 20 µL of the MuSC cell suspension was injected into their TA muscles. Each TA muscle received MuSCs from one shRNA condition (i.e., shCON, sh2, sh3, and sh2/3) in a random fashion for a total of 8 replicates per condition across 16 recipient mice.

For secondary transplantation, the TA muscles of primary transplant recipient mice were harvested and digested as described in the primary mouse MuSC isolation section. The cell suspension resulting from each TA muscle was evenly divided and injected into two recipient TA muscles for a total of 8 replicates per condition across 16 recipient mice.

### In vitro luciferase assay

To determine the effect of shRNA conditions on MuSC proliferation after 7 days in culture, primary MuSCs from CMV-*luc* mice were incubated in fresh growth medium containing 0.15 mg/mL D-Luciferin (Gold Biotechnology, St. Louis, MO, USA) for 15 min at 37 °C prior to measurement on a SpectraMax M3 plate reader (Molecular Devices, San Jose, CA, USA).

### In vivo bioluminescent imaging

In vivo bioluminescent imaging was used to track MuSC behavior after transplantation. Transplant recipient mice were anesthetized using isoflurane and injected with 125 µL of 30 mg/mL D-Luciferin prior to imaging (Gold Biotechnology, St. Louis, MO, USA). Bioluminescent imaging was performed using the IVIS^®^ Spectrum in vivo imaging system (PerkinElmer, Waltham, MA, USA). Digital images were obtained 12 min after injections and analyzed using Living Image Software (PerkinElmer, Waltham, MA, US). The engraftment threshold was set at 50,000 photons per second. Bioluminescent images were taken at 3, 11, 17, 25, 31, and 40 days after primary transplantation. For secondary transplantation experiments, bioluminescent images were taken 35 days after transplantation.

### Confluence analysis

Confluence measurements were performed as previously described^45^. Briefly, confluence was determined as the area of the cell culture surface covered by cells as measured by the Celigo S Imaging Cytometer (Nexcelom Bioscience, Lawrence, MA, USA).

### Statistics

Statistical analyses for experimental data were performed in GraphPad (Version 1.0.136) with either two-tailed unpaired *t* tests or one-way analysis of variance (ANOVA). Data are displayed as means ± standard deviation. Statistical significance was determined at *P* < 0.05. When a main effect of a one-way ANOVA was found to be statistically significant, a Tukey–Kramer post hoc test was conducted.

## Supplementary information

Supplementary Figure Legends

Supplementary Figure 2

Supplementary Figure 1

Supplementary Table 1

Supplementary Table 2

Supplementary Table 3

Supplementary Table 4

Supplementary Table 5

Supplementary Table 6

Supplementary Table 7

Supplementary Table 8

Supplementary Table 9

Supplementary Table 10

## Data Availability

All RNA-seq and MeRIP-seq data generated or analyzed for the present study are included in this article (including the accompanying Supplementary Tables) and have been deposited in the NCBI Gene Expression Omnibus data base (GSE144885).
