# Supplementary material for: A defined N6-methyladenosine (m6A) profile conferred by METTL3 regulates muscle stem cell/myoblast state transitions
Source: Cell Death Discov. 2020 Sep 29;6:95. doi: 10.1038/s41420-020-00328-5 (PMC7524727; doi:10.1038/s41420-020-00328-5)
Supplement: Supplementary file 1 — Supplementary Figure Legends [file 41420_2020_328_MOESM1_ESM.docx]

**Supplementary Figure 1.** m^6^A-modifications decrease in primary mouse myoblasts during differentiation

1. Isolated mRNA from primary mouse myoblasts in GM had greater global m^6^A-modification levels than mRNA isolated in 3d DM as measured by LC-MS (n=3). ***P<0.001.

**Supplementary Figure 2.** *Mettl3* knockdown reduces primary mouse MuSC confluence

1. Primary mouse MuSCs were isolated from CMV-*luc* mice, treated with shRNAs targeting *Mettl3* or a non-mammalian control sequence, and cultured for 7 days prior to measuring percent confluence (n=4). *P<0.05.
