# Supplementary figures and images for: A defined N6-methyladenosine (m6A) profile conferred by METTL3 regulates muscle stem cell/myoblast state transitions
Source: Cell Death Discov. 2020 Sep 29;6:95. doi: 10.1038/s41420-020-00328-5 (PMC7524727; doi:10.1038/s41420-020-00328-5)

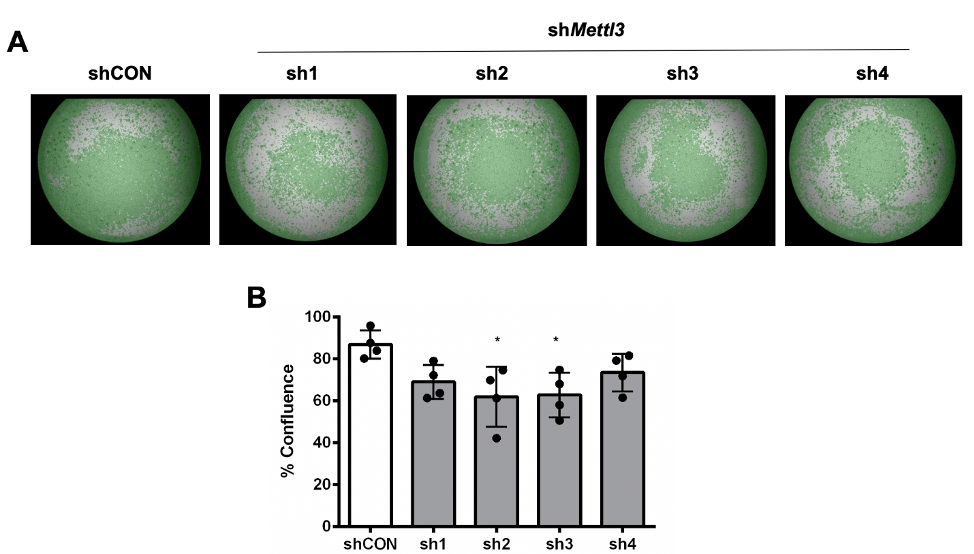

Supplement: Supplementary file 2 — Supplementary Figure 2 [file 41420_2020_328_MOESM2_ESM.png]

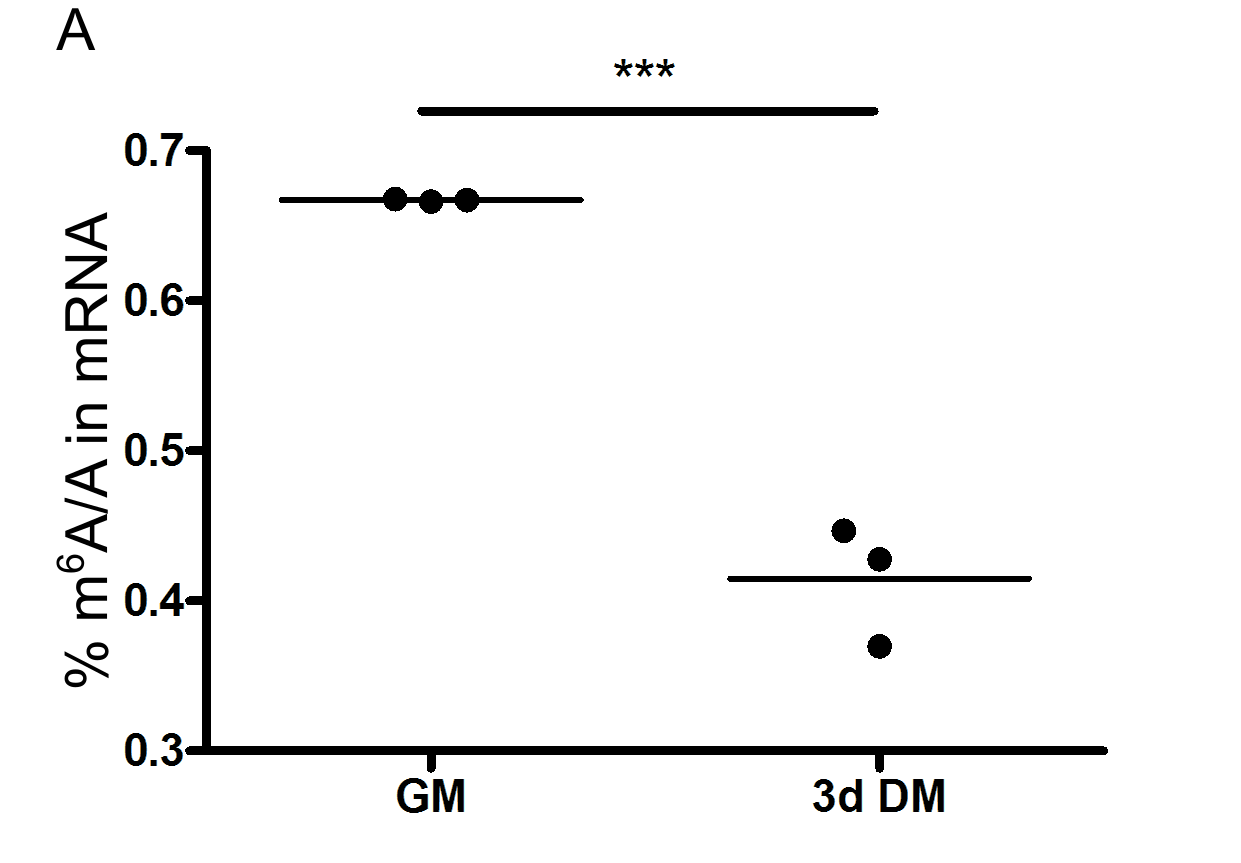

Supplement: Supplementary file 3 — Supplementary Figure 1 [file 41420_2020_328_MOESM3_ESM.png]
